# Bullying experiences before and after the transition from lower to upper secondary school: associations with subsequent mental health in a Swedish cohort

**DOI:** 10.1186/s12889-023-17443-4

**Published:** 2024-01-02

**Authors:** Sara Brolin Låftman, Karina Grigorian, Andreas Lundin, Viveca Östberg, Jonas Raninen

**Affiliations:** 1 Department of Public Health Sciences, Stockholm University, Stockholm, SE-106 91 Sweden; 2 Centre for Epidemiology and Community Medicine, Region Stockholm, Box 45436, Stockholm, SE-104 31 Sweden; 3 Department of Global Public Health, Karolinska Institutet, Stockholm, SE-171 77 Sweden; 4 Department of Clinical Neuroscience, Karolinska Institutet, Stockholm, SE-171 77 Sweden; 5 Centre for Alcohol Policy Research, La Trobe University, Melbourne, Australia

**Keywords:** Bullying, Victimisation, Depression, Anxiety, Mental health, Adolescents, School, Longitudinal, Prospective

## Abstract

**Background:**

Previous research has shown that exposure to bullying is linked to long-term adverse mental health consequences. However, prospective studies examining the persistence of bullying, using information from repeated time points, are limited. The aim of this study was to examine, firstly, the extent to which exposure to bullying among adolescents in Sweden changes between grades 9 (age 15–16) and 11 (age 17–18) (i.e., before and after the transition from lower to upper secondary school); secondly, whether being bullied in grade 9 or 11 is associated with depression and anxiety symptoms at age 20–21; and thirdly, if being bullied in both grade 9 and 11 is linked to an even higher likelihood of subsequent depression and anxiety symptoms. Potential differences by gender were investigated throughout.

**Methods:**

Data was derived from the Swedish cohort study Futura01 involving individuals attending grade 9 in the school year 2016/17 (*n* = 2323). We utilised self-reported information from three survey waves conducted in 2017, 2019, and 2022, and linked registry information on sociodemographic characteristics. Bullying was assessed using a single item in waves 1 and 2. Depression and anxiety symptoms were measured using the Patient Health Questionnaire-4 (PHQ-4) in wave 3. Gender stratified binary logistic regressions were performed.

**Results:**

Among those who were bullied in grade 9, 22.6% of males and 35.8% of females continued to experience bullying in grade 11. For females, exposure to bullying in grade 9 or 11 was associated with an increased likelihood of reporting depression and anxiety symptoms at age 20–21, with the highest odds for those bullied at both time points. For males, only one statistically significant association was identified – specifically, between being bullied in grade 9 and subsequent depression symptoms.

**Conclusions:**

For a majority of adolescents who experience bullying in lower secondary school, but not all, the transition to upper secondary school proves to be beneficial as the bullying typically does not persist. However, bullying can have long-term health effects, in particular for females. These findings emphasise the importance of effective measures to address bullying within schools.

**Supplementary Information:**

The online version contains supplementary material available at 10.1186/s12889-023-17443-4.

## Background

A growing number of studies have demonstrated that exposure to bullying at school is associated with long-term adverse mental health consequences [1–10]. Bullying is widely acknowledged as a major stressor [11], and its detrimental effects on health are mediated through various mechanisms. In the school context, belonging to peer groups plays an important role in influencing self-esteem and identity. Conversely, experiencing bullying signals that the individual does not adhere to the group standards. Being exposed to bullying can thus be characterised as a social evaluative threat [10–12]. As a result of continuous negative evaluations from others, individuals who are bullied can develop a negative self-image [10], decreased self-esteem [13], as well as reduced self-efficacy [14]. Additionally, adolescents who experience bullying may receive diminished social support [14], indicating less supportive relationships with both peers [10, 15, 16] and adults [10, 16]. Consequently, individuals who are bullied are less likely to benefit from both the positive direct effects of social support on health and from its potential as a stress buffer [17]. Inadequate relationships with parents and teachers may also contribute to many adolescents not disclosing their experiences of bullying [16], thereby hindering potential efforts to halt the bullying.

Prospective studies that investigate the persistence of bullying in relation to subsequent mental health are currently limited. Existing studies suggest an exposure-response pattern, indicating that a continuous experience of being bullied is more strongly associated with later adverse mental health. In their study based on Danish data from the West Jutland Cohort Study, Winding et al. [18] demonstrated that individuals who were bullied at age 15 or 18 had a higher likelihood of reporting depressive symptoms at age 28, with the highest risk observed among those who experienced bullying at both time points. Similarly, in their analyses of data from the British Avon Longitudinal Study of Parents and Children (ALSPAC), Zwierzynska et al. [19] showed that children who were bullied at both age 8 and 10 were more likely to report depression symptoms in mid-adolescence compared with those who were bullied at only one of the time points. However, further research on the role of continuous experiences of bullying victimisation is warranted. Moreover, although it has been shown that school transitions are associated with a decline in bullying victimisation [20, 21], studies from different contexts are relevant.

Some previous studies have reported prospective links between exposure to bullying and later adverse mental health outcomes only for females [1, 10], pointing to the relevance of performing gender-stratified analyses. Additionally, prior research has demonstrated that both the risk of being bullied and mental health may vary by sociodemographic characteristics. Specifically, studies have highlighted associations between family type and bullying [22] as well as mental health [23]. The family’s socioeconomic position has also been shown to be associated with both bullying [24] and mental health [25]. Additionally, parental country of birth has been demonstrated to be linked with bullying [26] and mental health [27]. Therefore, these factors should be taken into consideration in any analysis of bullying and mental health. Furthermore, while we hypothesise that exposure to bullying leads to poorer mental health, it is important to note that psychological problems have also been shown to predict the likelihood of being bullied [28, 29]. Therefore, for a more accurate modeling of the association between bullying and subsequent mental health outcomes, and to better discern the temporal order between them, it is essential to control for prior mental health status as a confounding factor [19, 30].

### Aim of the study

The aim of the current study was to examine, firstly, the extent to which exposure to bullying among adolescents in Sweden changes between grades 9 (age 15–16) and 11 (age 17–18) (i.e., before and after the transition from lower to upper secondary school); secondly, if being bullied in grade 9 or 11 is associated with depression and anxiety symptoms at age 20–21; and thirdly, if being bullied in both grade 9 and 11 is linked with an even higher likelihood of reporting subsequent depression and anxiety symptoms. Potential differences by gender were investigated throughout.

## Methods

### Data material

The data was derived from Futura01, a national Swedish cohort study of adolescents attending grade 9 in the school year 2016/17 (age 15–16 years). The first wave was carried out as a classroom questionnaire in 2017, when participants attended the ninth and final grade of lower secondary school (*n* = 5537). The second wave was performed in 2019 when respondents typically attended the second grade of upper secondary school (grade 11; age 17–18 years) as a web survey or postal survey (*n* = 4141). The third wave was performed in 2022 after the participants had finished upper secondary school (age 20–21 years) as a web survey (*n* = 3193). All three waves were collected in the spring. The number of individuals who participated in all three surveys was 2836. The present study includes information from those who participated in all three waves with non-missing information on the study variables (*n* = 2323; of whom 966 males and 1357 females). The exclusion of participants was largely due to those who answered “don’t know” or skipped the question on bullying (wave 1: *n* = 479 and *n* = 57, respectively; wave 2: *n* = 213 and *n* = 255, respectively). Ethical approval was obtained from the Swedish Ethical Review Authority (ref. 2021-06504-01; 2022-02781-02; 2022-06502-02).

### Measures

Exposure to bullying was measured in waves 1 and 2 by the question “Have you been bullied during the past 12 months?” with the response categories “No”, “Yes”, and “Don’t know”. Participants who answered “Don’t know” were coded as missing.

Depression symptoms and anxiety symptoms over the last two weeks were measured in wave 3 by the Patient Health Questionnaire-4 (PHQ-4) [31, 32]. This battery includes the Patient Health Questionnaire-2 (PHQ-2) with two items on depression symptoms [33] and the Generalized Anxiety Disorder-2 (GAD-2) with two items on anxiety symptoms [34]. For both measures, we used a cutoff at ≥ 3 [32].

Gender was based on information from the participants’ personal security numbers. While this information indicates biological sex rather than socially constructed gender, we posit that the associations between sex/gender and the variables under study are largely influenced by social rather than biological factors. Therefore, we use the term ‘gender’.

Covariates included family type which was measured from survey information in wave 1; parental education (measuring the highest educational level among parents) and parental country of birth derived from registry information; and medication for depression (e.g., Fluoxetin, Oralin, Zoloft) and anxiety (e.g., Theralen, Sobril, Oralin) which was based on survey information from waves 1 and 2.

### Statistical analysis

The change in exposure to bullying between grade 9 and grade 11 was examined by cross-tabulations in the total sample and separately for males and females. To examine the associations between exposure to bullying and subsequent depression and anxiety symptoms, we first performed cross-tabulations with chi square tests in the total sample and separately for males and females. Next, we carried out gender-stratified binary logistic regression analyses with depression and anxiety symptoms as the dependent variables and exposure to bullying as the independent variable. All regression analyses adjusted for the full set of covariates. In addition, we performed analyses of the total sample and to assess differences by gender, we included interactions between exposure to bullying and gender. Wald tests were performed, comparing the model fit between models with and without the interaction terms. Due to the study design, with students nested in classes at baseline, robust errors were estimated clustering at the class level. The estimates presented are odds ratios (OR) with 95% confidence intervals (95% CI). All analyses were performed in Stata, version 17 [35].

### Large Language models (LLM)

ChatGPT was utilised for proofreading and language editing of the manuscript.

## Results

Descriptives are presented in Table 1, for the total study sample and stratified by gender. Differences by gender were investigated with chi square tests. In the study sample, 6.4% of males and 8.0% of females reported being bullied in grade 9, while 4.2% of males and 6.2% of females reported being bullied in grade 11. Combining information from the two time points revealed that among males, 5.0% reported being bullied only in grade 9, 2.8% only in grade 11, and 1.4% in both grades 9 and 11. For females, the corresponding proportions were 5.2%, 3.3%, and 2.9%, respectively. At age 20–21, 22.9% of males and 26.0% of females reported depression symptoms, while 16.4% of males and 32.7% of females reported anxiety symptoms. Descriptives of the study variables in the full t1 sample are displayed in the Supplementary Material, Table S1. Comparison of Table 1 and Table S1 shows that students who reported being bullied in grade 9 were less likely to take part in the follow-up surveys. There was also systematic bias in the attrition with somewhat higher dropout among males, students not living with two original parents, students whose parents had less than tertiary education, students with two parents born abroad, and students who used medication for depression and anxiety.

**Table 1:** Descriptives of the study variables in the total study sample and by gender, and χ^2^ tests of differences by gender

	All (*n* = 2323)		Males (*n* = 966)		Females (*n* = 1357)		χ^2^
	n	%	n	%	n	%	
Bullied in grade 9 (age 15–16)							
No	2152	92.6	904	93.6	1248	92.0	
Yes	171	7.4	62	6.4	109	8.0	2.16
Bullied in grade 11 (age 17–18)							
No	2198	94.6	925	95.8	1273	93.8	
Yes	125	5.4	41	4.2	84	6.2	4.20*
Bullied							
Neither in grade 9 nor 11	2080	89.5	877	90.8	1203	88.6	
In grade 9 only	118	5.1	48	5.0	70	5.2	
In grade 11 only	72	3.1	27	2.8	45	3.3	
In both grade 9 and 11	53	2.3	14	1.4	39	2.9	5.84
Depressive symptoms (age 20–21)							
No	1749	75.3	745	77.1	1004	74.0	
Yes	574	24.7	221	22.9	353	26.0	2.98
Anxiety symptoms (age 20–21)							
No	1721	74.1	808	83.6	913	67.3	
Yes	602	25.9	158	16.4	444	32.7	78.70***
Family type							
Two original parents	1669	71.8	725	75.0	944	69.6	
One parent	291	12.5	93	9.6	198	14.6	
Shared residence	315	13.6	131	13.6	184	13.6	
Other	48	2.1	17	1.8	31	2.3	14.21**
Parental education							
≤ 2 years secondary or less	323	13.9	115	11.9	208	15.3	
≥ 3 years secondary	435	18.7	179	18.5	256	18.9	
Tertiary	1565	67.4	672	69.6	893	65.8	5.97
Parental country of birth							
At least one in Sweden	1972	84.9	830	85.9	1142	84.2	
Two parents outside Sweden	351	15.1	136	14.1	215	15.8	1.37
Medication for depression	102	4.4	23	2.4	79	5.8	15.91***
Medication for anxiety	127	5.5	19	2.0	108	8.0	39.20***

***p < 0.001 **p < 0.01 *p < 0.05

**Fig. 1:**
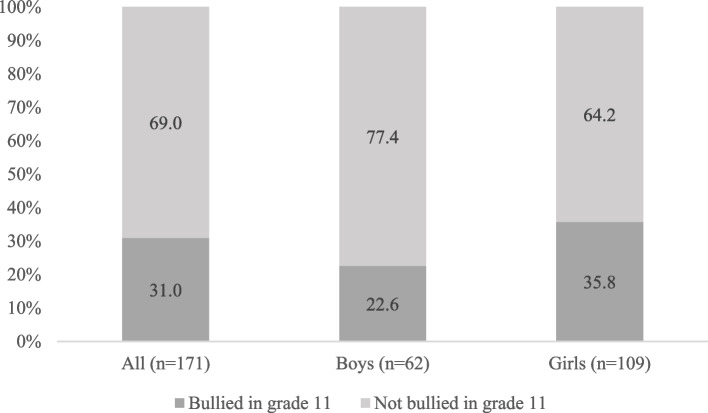
Proportions of adolescents who were bullied in grade 9, who reported to be bullied or not bullied in grade 11, for all and separately for boys and girls

As shown in Fig. 1, among those who were bullied in grade 9, 22.6% of males and 35.8% of females continued to be bullied also in grade 11 (the gender difference was not statistically significant; data not presented).

Table 2 presents cross-tabulations between exposure to bullying in grade 9 and 11 and depression and anxiety symptoms at age 20–21, and results from chi square tests assessing differences between groups. In the total sample, depression and anxiety symptoms at age 20–21 were more common among those who were bullied in grade 9 and in grade 11, respectively. Depression and anxiety symptoms at age 20–21 were least common among those were bullied at neither time point, more common among those who were bullied either in grade 9 or in grade 11, and most frequent among those who were bullied at both time points. For males, none of the differences between groups was statistically significant. For females, however, there were clear and statistically significant differences between groups. Depression and anxiety symptoms at age 20–21 were more common among females who reported to be bullied in grade 9 and in grade 11. There were also statistically significant associations between exposure to bullying at one or two time points and subsequent depression and anxiety symptoms (although the difference in depression symptoms between females who were bullied only in grade 11 and those who were bullied in both grades 9 and 11 was minor). Additional descriptive analyses including participants who replied “Don’t know” or with missing information on the questions about bullying are displayed in the Supplementary Material, Table S2. These findings indicate that, generally, participants who responded “don’t know” or skipped the question on bullying were more likely to report subsequent symptoms of depression and anxiety compared to those who stated that they were not bullied.

**Table 2:** Crosstabulations between exposure to bullying in grades 9 (age 15–16) and 11 (age 17–18) and depression and anxiety symptoms at age 20–21, and χ^2^ tests of differences between groups

	All				Males				Females			
	Depression symptoms (age 20–21)		Anxiety symptoms (age 20–21)		Depression symptoms (age 20–21)		Anxiety symptoms (age 20–21)		Depression symptoms (age 20–21)		Anxiety symptoms (age 20–21)	
	% (n)	χ^2^	% (n)	χ^2^	% (n)	χ^2^	% (n)	χ^2^	% (n)	χ^2^	% (n)	χ^2^
Bullied in grade 9 (age 15–16)												
No	23.8 (511)		24.9 (536)		22.2 (201)		15.9 (144)		24.8 (310)		31.4 (392)	
Yes	36.8 (63)	14.61***	38.6 (66)	15.46***	32.3 (20)	3.30	22.6 (14)	1.88	39.5 (43)	11.12**	47.7 (52)	12.09**
Bullied in grade 11 (age 17–18)												
No	23.7 (520)		25.1 (552)		22.4 (207)		16.2 (150)		24.6 (313)		31.6 (402)	
Yes	43.2 (54)	24.28***	40.0 (50)	13.65***	34.2 (14)	3.08	19.5 (8)	0.31	47.6 (40)	21.72***	50.0 (42)	12.15***
Bullied												
Neither in grade 9 nor 11	23.2 (482)		24.5 (509)		22.0 (193)		15.6 (137)		24.0 (289)		30.9 (372)	
In grade 9 only	32.2 (38)		36.4 (43)		29.2 (14)		27.1 (13)		34.3 (24)		42.9 (30)	
In grade 11 only	40.3 (29)		37.5 (27)		29.6 (8)		25.9 (7)		46.7 (21)		44.4 (20)	
In both grade 9 and 11	47.2 (25)	29.95***	43.4 (23)	22.54***	42.9 (6)	5.32	7.1 (1)	7.06	48.7 (19)	25.38***	56.4 (22)	17.79***

***p < 0.001 **p < 0.01 *p < 0.05

Next, we performed a series of gender-stratified binary logistic regression analyses predicting the likelihood of reporting depression and anxiety symptoms at age 20–21 by prior exposure to bullying, controlling for family type, parental education, parental country of birth, and prior medication for depression and anxiety (Table 3). Reflecting the cross-tabulations, for males, there were no statistically significant differences between exposure to bullying and later depression symptoms. However, an increased risk of anxiety symptoms was observed in males who were bullied in grade 9 only (OR 2.07, 95% CI 1.01–4.24). For females, there were clear associations between exposure to bullying and both depression and anxiety symptoms, even when adjusting for the covariates. Compared with females who were bullied at neither time point, increased risks of depression symptoms were seen among those who were bullied at in grade 11 only (OR 2.34, 95% CI 1.27–4.30) and among those who were bullied at two time points (OR 2.40, 95% CI 1.18–4.86). The difference between the two latter categories was not statistically significant (data not presented). With regards to anxiety symptoms, the analyses showed a clearer graded association with exposure to bullying. Compared with females who were bullied at neither time point, increased risks of anxiety symptoms were seen among those bullied in grade 9 only (OR 1.64, 95% CI 1.01–2.66) and more so among those bullied at two time points (OR 2.37, 95% CI 1.18–4.77). However, the difference between these two categories was not statistically significant (data not presented).

**Table 3:** Results from binary logistic regression analyses predicting depression and anxiety symptoms at age 20–21 by exposure to bullying in grades 9 (age 15–16) and 11 (age 17–18). Models fully adjusted for bullying, family type, parental education, parental country of birth, and medication for depression and anxiety

	Males (*n* = 966)				Females (*n* = 1357)			
	Depression symptoms		Anxiety symptoms		Depression symptoms		Anxiety symptoms	
	OR	95% CI	OR	95% CI	OR	95% CI	OR	95% CI
Bullied								
Neither in grade 9 nor 11 (ref.)	1.00	-	1.00	-	1.00	-	1.00	-
In grade 9 only	1.55	0.82–2.94	2.07*	1.01–4.24	1.64	0.96–2.82	1.64*	1.01–2.66
In grade 11 only	0.99	0.42–2.34	1.32	0.53–3.29	2.34**	1.27–4.30	1.50	0.80–2.82
In both grade 9 and 11	2.67	0.88–8.09	0.37	0.05–2.85	2.40*	1.18–4.86	2.37*	1.18–4.77
Family type								
Two original parents (ref.)	1.00	-	1.00	-	1.00	-	1.00	-
One parent	1.96**	1.20–3.20	1.98*	1.17–3.36	1.37	0.98–1.92	1.32	0.94–1.87
Shared residence	1.40	0.88–2.22	1.48	0.90–2.43	0.96	0.67–1.37	1.32	0.94–1.84
Other	2.01	0.70–5.78	0.67	0.16–2.71	1.48	0.71–3.06	2.04*	1.02–4.08
Parental education								
≤ 2 years secondary or less	1.15	0.65–2.04	1.07	0.58–1.97	1.23	0.79–1.91	1.28	0.84–1.94
≥ 3 years secondary (ref.)	1.00	-	1.00	-	1.00	-	1.00	-
Tertiary	1.13	0.73–1.73	1.02	0.65–1.58	0.88	0.62–1.24	1.07	0.78–1.47
Parental country of birth								
At least one in Sweden (ref.)	1.00	-	1.00	-	1.00	-	1.00	-
Two parents outside Sweden	2.11**	1.37–3.24	2.20**	1.37–3.53	1.48*	1.05–2.08	1.18	0.84–1.66
Medication for depression	2.84*	1.03–7.87	1.44	0.51–4.05	2.51**	1.26–5.01	1.54	0.83–2.84
Medication for anxiety	1.86	0.61–5.66	0.83	0.24–2.84	1.28	0.71–2.32	2.09**	1.20–3.63

**p < 0.01 *p < 0.05

As to the covariates, for males, living with one parent was associated with a higher likelihood of reporting both depression (OR 1.96, 95% CI 1.20–3.20) and anxiety symptoms (OR 1.98, 95% CI 1.17–3.36) compared with those living with two original parents. Furthermore, parental country of birth showed a statistically significant association with the outcomes, indicating that males with two parents born outside Sweden reported a higher likelihood of both depression (OR 2.11, 95% CI 1.37–3.24) and anxiety symptoms (OR 2.20, 95% CI 1.37–3.53) compared with those with at least one Swedish-born parent. Prior medication for depression was associated with a greater likelihood of depression symptoms at t3 (OR 2.84, 95% CI 1.03–7.87). For females, living in an “other” family type was associated with a higher likelihood of reporting anxiety symptoms (OR 2.04, 95% CI 1.02–4.08). Females with two parents born abroad were more likely to report depression symptoms compared with those with at least one parent born in Sweden (OR 1.48, 95% CI 1.05–2.08). Prior medication for depression was associated with a greater likelihood of depression symptoms at t3 (OR 2.51, 95% CI 1.26–5.01). Similarly, prior medication for anxiety was associated with a greater likelihood of anxiety symptoms at t3 (OR 2.09, 95% CI 1.20–3.63).

Finally, we performed binary logistic regression analyses using the total sample, with results presented in the Supplementary Material, Table S3. We also included interaction terms between exposure to bullying and gender, which were evaluated through Wald tests. These analyses showed that the associations between exposure to bullying and depression and anxiety symptoms did not differ significantly between males and females (depression symptoms: χ^2^ = 2.28, *p* = 0.516; anxiety symptoms: χ^2^ = 2.99, *p* = 0.393).

## Discussion

The purpose of this study was to examine the extent to which exposure to bullying among adolescents in Sweden changes between grades 9 and 11. Additionally, it aimed to investigate whether being bullied in grade 9 or 11 is associated with an increased likelihood of reporting depression and anxiety symptoms at age 20–21. Furthermore, the study explored whether being bullied in both grade 9 and 11 is linked with an even higher likelihood of subsequent depression and anxiety symptoms. Gender differences were examined throughout the study.

The findings showed a decline in bullying victimisation between grades 9 and 11. Of those who reported to be bullied in grade 9, a minority (22.6% of males and 35.8% of females) continued to experience bullying also in grade 11. These results align with findings from previous studies suggesting that for a majority of those who experience bullying, but not all, school transitions are positive as the bullying does not persist [20, 21]. The decline in bullying can be attributed to both developmental (age-related) processes and contextual factors associated with changes in social settings. Notably, a study by Wang et al. [20] utilised a naturally occurring experiment where some students transitioned into a new school between grades 5 and 6 while others remained in the same school. The study indicated that the change in context was linked to a decrease in bullying victimisation, although this association was only observed in girls. The authors concluded that changes in peer victimisation may be understood as contextual processes [20].

The analyses revealed distinct associations between bullying and later development of depression and anxiety symptoms among females. However, no such associations were found among males, except for a statistically significant link between being bullied in grade 9 and subsequent anxiety symptoms. The highest odds of depression and (especially) anxiety symptoms were observed in females who experienced bullying at both time points. These findings in females align with previous research, suggesting a prospective link between exposure to bullying and poorer mental health [1–10]. Our findings also add to the limited amount of earlier studies which demonstrate that continuous exposure to bullying is particularly strongly associated with adverse mental health outcomes [18, 19]. Severe stress is one key mechanism in the association between exposure to bullying and later depression and anxiety symptoms [11]. Experiencing bullying, in terms of being constantly negatively evaluated by peers, is also likely to lead to an internalisation of negative evaluations in terms of poorer self-image [10] as well as poorer self-esteem [13] and lower self-efficacy [14], which may in turn be linked to depression and anxiety. Furthermore, adolescents who are bullied tend to have lower levels of social support [10, 14]. Prior studies have shown that adolescents who are bullied do not only have fewer friends [15], but also poorer relations with parents as well as teachers [10, 16]. This implies that adolescents who experience bullying are less likely to benefit both from the direct and the stress-buffering effects of social support [17].

While the relative lack of statistically significant associations among males could partly be due to limited statistical power, also this result is consistent with findings from previous studies. Östberg et al. [10] documented links between exposure to bullying at age 10–18 and adverse psychological health ten years later for females but not for males. Sourander et al. [1] showed that girls who were bullied at age 8 had an increased likelihood of subsequent psychiatric hospital and psychopharmacologic treatment, whereas no such association was found for boys when controlling for earlier psychopathology. Our findings also align with a study by Modin et al. [36] which showed that a lower status position in school was associated with higher risks of hospital admissions due to anxiety and depression in adulthood among females, but not among males. One interpretation of these findings as a whole is that females may on average be more affected by strained relations and by negative evaluations from others, compared with males [10]. Indeed, as discussed by Rudolph [37], girls tend to perceive negative family and peer relations as more stressful than boys, leading to heightened negative emotional responses to interpersonal stress, including depression and anxiety. One possible explanation is that girls may have a greater psychological and emotional investment in interpersonal success than boys. Therefore, it is plausible that girls, compromising their self-view through lower self-esteem, are more sensitive to negative evaluations by peers compared to boys [37]. Another possible explanation for gender differences could be linked to social norms causing girls and boys to encounter distinct forms of bullying and face varying expectations on how to behave and which coping strategies to employ [38]. This, in turn, may lead to distinct mental health consequences for boys and girls. Another possibility is that bullying is more clearly associated with other types of health outcomes for males, and/or that the health effects become visible at later ages [10].

### Strengths and limitations

The main strength of the current study is the utilisation of a nationally representative sample and self-reported information on bullying from two time points. The use of PHQ-4 which is a validated measure of depression and anxiety symptoms, reflecting two of the most common psychiatric conditions in young adults in Sweden [39], is also a merit. Another benefit is the linkage to registry data, enabling us to adjust for relevant sociodemographic background characteristics.

Nonetheless, there are also limitations. One drawback is the crude measure of bullying, based on only one question and without any definition of the concept in the questionnaire. Indeed, it has been suggested that using the term ‘bullying’ without a clear definition can be problematic, as it is not universally perceived in the same way by all adolescents. Accordingly, the various methods researchers employ to measure bullying make comparisons of prevalence difficult [40]. This is the true also for studies conducted in Sweden [41]. The Swedish Health Behaviour in School-aged Children (HBSC) study employs a question on exposure to bullying that includes a clear definition, albeit with a shorter time frame than in Futura01. According to the Swedish HBSC study of 2017/18, approximately 7% of boys and 6% of girls in grade 9 reported being bullied at least 2 or 3 times a month during the past months [42], i.e., a slightly lower prevalence than in the current study (see Table S1). Nonetheless, considering the different time frames, with the HBSC referring to the past months and Futura01 referring to the past year, the difference in prevalence between the two studies seems reasonable.

An important shortcoming of our measure is however the high proportions of (especially) “don’t know” and missing answers to the questions on bullying. Descriptive analyses were conducted, considering these two categories as well. In many cases, participants who marked “don’t know” or skipped the bullying questions were more likely to report subsequent depression and anxiety symptoms compared to those who stated they were not bullied. This might be related to the absence of a clear definition of bullying, leading to uncertainty among respondents, some of whom may have experienced bullying-like acts.

Another limitation revolves around the fact that despite the large sample there was a rather small number of participants who reported to be bullied at both time points (males: *n* = 14; females: *n* = 39), which implied a lack of statistical power in the analyses of males. Furthermore, to account for prior mental health, which may be a confounder, we controlled for self-reported measures of medication for depression and anxiety in grade 9 and 11. However, since a substantial proportion of young persons do not seek and receive care for their mental health conditions [43], and since other types of treatment such as psychological therapy are also common [44], these measures were not optimal proxies for depression and anxiety symptoms. Analyses of t3 data (not presented) showed that among those classified with depression symptoms according to PHQ-2, 18.6% reported to take medication for depression at t3; and among those classified with anxiety symptoms according to GAD-2, 18.8% reported to take medication for anxiety at t3.

It should also be acknowledged that we did not control for other potential confounders that may be associated with both bullying and mental health problems, such as parental mental illness and other indicators of childhood adverse experiences. Therefore, despite identifying temporal associations between exposure to bullying and subsequent mental health problems, the likely presence of omitted variables bias prevents us from drawing any firm conclusions about causality.

Another limitation concerned the attrition across waves, which may have compromised the generalisability of our findings. Importantly, as observed in the comparison of the descriptives of the study sample and the full baseline sample, students who reported being bullied in grade 9 were somewhat less likely to participate in the follow-up surveys. In addition, since some studies have shown that school absenteeism is more common among students who are bullied [45], it is possible that adolescents who were bullied at school were also less likely to participate in the study at all, since the baseline survey was conducted at school. Nonetheless, these biases are probably more likely to have underestimated our results rather than the other way around.

Lastly, it should be highlighted that the data were collected in Sweden, a country with relatively low rates of bullying in an international perspective [46], albeit with increasing levels in recent years, as noted by Bjereld et al. [41]. Therefore, the scope of generalisability to other national contexts may be limited.

## Conclusions

The current study contributes to the growing body of research demonstrating the long-term health consequences of exposure to bullying. Additionally, the study suggests that continuous exposure to bullying may be particularly detrimental. These findings underscore the importance of implementing effective measures against bullying in schools. This could involve the adaptation of evidence-based anti-bullying programmes, as well as other strategies that foster a positive school climate. For example, reducing teachers’ time pressure and work-related stress may provide them with better opportunities to intervene in bullying situations [47]. From a broader perspective, it should be emphasised that a school climate free from bullying can benefit the mental health of not only those who are directly exposed, but all students [12, 13, 48].

### Supplementary Information

**Additional file 1: Table S1.** Descriptives of the study variables in the full t1 sample. **Table S2.** Crosstabulations between exposure to bullying (displaying the categories no/yes/don’t know/missing) in grades 9 (age 15-16) and 11 (age 17-18) and depression and anxiety symptoms at age 20-21, and χ^2^ tests of differences between groups. **Table S3.** Results from binary logistic regression analyses predicting depressive and anxiety symptoms at age 20-21 by exposure to bullying in grades 9 (age 15-16) and 11 (age 17-18). Models fully adjusted for bullying, gender, family type, parental education, parental country of birth, and medication for depression and anxiety. Wald tests from separate models that include interaction terms between bullied and gender. *n*=2323.

## Data Availability

The data used for the current study are available from Karolinska Institutet but restrictions apply to the availability of these data, and are therefore not publicly available. Data are however available from the Principal Investigator Dr. Jonas Raninen (jonas.raninen@ki.se) upon reasonable request and with permission of Karolinska Institutet and ethical approval from the Swedish Ethical Review Authority.
