# Supplementary material for: Bullying experiences before and after the transition from lower to upper secondary school: associations with subsequent mental health in a Swedish cohort
Source: BMC Public Health. 2024 Jan 2;24:27. doi: 10.1186/s12889-023-17443-4 (PMC10762947; doi:10.1186/s12889-023-17443-4)
Supplement: Supplementary file 1 — Additional file 1: Table S1. Descriptives of the study variables in the full t1 sample. Table S2. Crosstabulations between exposure to bullying (displaying the categories no/yes/don’t know/missing) in grades 9 (age 15-16) and 11 (age 17-18) and depression and anxiety symptoms at age 20-21, and χ2 tests of differences between groups. Table S3. Results from binary logistic regression analyses predicting depressive and anxiety symptoms at age 20-21 by exposure to bullying in grades 9 (age 15-16) and 11 (age 17-18). Models fully adjusted for bullying, gender, family type, parental education, parental country of birth, and medication for depression and anxiety. Wald tests from separate models that include interaction terms between bullied and gender. n=2323. [file 12889_2023_17443_MOESM1_ESM.docx]

Supplementary Material. Table S1. Descriptives of the study variables in the full t1 sample.

.

|  | All  (n=5537) | | Males (n=2743) | | Females (n=2794) | | χ^2^ |
| --- | --- | --- | --- | --- | --- | --- | --- |
|  | n | % | n | % | n | % |  |
| Bullied in grade 9 (age 15-16) |  |  |  |  |  |  |  |
| No | 4546 | 90.9 | 2280 | 91.6 | 2266 | 90.2 |  |
| Yes | 455 | 9.1 | 208 | 8.4 | 247 | 9.8 | 3.26 |
| *Don’t know* | *479* | *-* | *225* | *-* | *254* | *-* |  |
| *Missing* | *57* | *-* | *30* | *-* | *27* | *-* |  |
|  |  |  |  |  |  |  |  |
| Bullied in grade 11 (age 17-18) |  |  |  |  |  |  |  |
| No | 3460 | 94.2 | 1553 | 95.3 | 1907 | 93.3 |  |
| Yes | 214 | 5.8 | 76 | 4.7 | 138 | 6.7 | 7.17** |
| *Don’t know* | *213* | *-* | *102* | *-* | *111* | *-* |  |
| *Missing* | *1650* | *-* | *1012* | *-* | *638* | *-* |  |
|  |  |  |  |  |  |  |  |
| Bullied |  |  |  |  |  |  |  |
| Neither in grade 9 nor 11 | 2971 | 88.6 | 1335 | 89.8 | 1636 | 87.7 |  |
| In grade 9 only | 193 | 5.8 | 84 | 5.7 | 109 | 5.8 |  |
| In grade 11 only | 113 | 3.4 | 45 | 3.0 | 68 | 3.7 |  |
| In both grade 9 and 11 | 74 | 2.2 | 22 | 1.5 | 52 | 2.8 | 7.81 |
| *Don’t know/missing* | *2186* | *-* | *1257* | *-* | *929* | *-* |  |
|  |  |  |  |  |  |  |  |
| Depressive symptoms (age 20-21) |  |  |  |  |  |  |  |
| No | 2461 | 74.3 | 1102 | 76.3 | 1359 | 72.7 |  |
| Yes | 853 | 25.7 | 342 | 23.7 | 511 | 27.3 | 5.65* |
| *Missing* | *2223* | *-* | *1299* | *-* | *924* | *-* |  |
|  |  |  |  |  |  |  |  |
| Anxiety symptoms (age 20-21) |  |  |  |  |  |  |  |
| No | 2430 | 73.2 | 1201 | 82.9 | 1229 | 65.7 |  |
| Yes | 890 | 26.8 | 247 | 17.1 | 643 | 34.3 | 124.40*** |
| *Missing* | *2217* | *-* | *1295* | *-* | *922* | *-* |  |
|  |  |  |  |  |  |  |  |
| Family type |  |  |  |  |  |  |  |
| Two original parents | 3734 | 67.4 | 1872 | 68.3 | 1862 | 66.6 |  |
| One parent | 823 | 14.9 | 345 | 12.6 | 478 | 17.1 |  |
| Shared residence | 784 | 14.2 | 413 | 15.1 | 371 | 13.3 |  |
| Other | 196 | 3.5 | 113 | 4.1 | 83 | 3.0 | 27.89*** |
| *Missing* | *0* | *-* | *0* | *-* | *0* | *-* |  |
|  |  |  |  |  |  |  |  |
| Parental education |  |  |  |  |  |  |  |
| ≤2 years secondary or less | 993 | 18.4 | 471 | 17.6 | 522 | 19.1 |  |
| ≥3 years secondary | 1159 | 21.4 | 575 | 21.5 | 584 | 21.4 |  |
| Tertiary | 3255 | 60.2 | 1627 | 60.9 | 1628 | 59.5 | 2.00 |
| *Missing* | *130* | *-* | *70* | *-* | *60* | *-* |  |
|  |  |  |  |  |  |  |  |
| Parental country of birth |  |  |  |  |  |  |  |
| At least one in Sweden | 4400 | 82.1 | 2204 | 83.0 | 2196 | 81.2 |  |
| Two parents outside Sweden | 958 | 17.9 | 451 | 17.0 | 507 | 18.8 | 2.86 |
| *Missing* | *179* | *-* | *88* | *-* | *91* | *-* |  |
|  |  |  |  |  |  |  |  |
| Medication for depression | 266 | 6.6 | 78 | 4.3 | 188 | 8.5 | 29.42*** |
| *Missing* | *1493* | *-* | *910* | *-* | *583* | *-* |  |
|  |  |  |  |  |  |  |  |
| Medication for anxiety | 324 | 8.0 | 79 | 4.3 | 245 | 11.1 | 62.27*** |
| *Missing* | *1481* | *-* | *905* | *-* | *576* | *-* |  |

Table S2. Crosstabulations between exposure to bullying (displaying the categories no/yes/don’t know/missing) in grades 9 (age 15-16) and 11 (age 17-18) and depression and anxiety symptoms at age 20-21, and χ^2^ tests of differences between groups.

|  | All | | | | Males | | | | Females | | | |
| --- | --- | --- | --- | --- | --- | --- | --- | --- | --- | --- | --- | --- |
|  | Depression symptoms  (age 20-21)  (n=3314) | | Anxiety  symptoms  (age 20-21)  (n=3320) | | Depression symptoms  (age 20-21)  (n=1444) | | Anxiety  symptoms  (age 20-21)  (n=1448) | | Depression symptoms  (age 20-21)  (n=1870) | | Anxiety  symptoms  (age 20-21)  (n=1872) | |
|  | %  (n) | χ^2^ | %  (n) | χ^2^ | %  (n) | χ^2^ | %  (n) | χ^2^ | %  (n) | χ^2^ | %  (n) | χ^2^ |
| Bullied in grade 9  (age 15-16) |  |  |  |  |  |  |  |  |  |  |  |  |
| No | 24.3  (667) |  | 24.8  (681) |  | 22.7  (273) |  | 15.8  (191) |  | 25.6  (394) |  | 31.8  (490) |  |
| Yes | 39.2  (103) |  | 39.6  (105) |  | 34.3 (37) |  | 29.4  (32) |  | 42.6  (66) |  | 46.8  (73) |  |
| Don’t know | 26.1  (74) |  | 34.7  (99) |  | 22.1  (27) |  | 18.9 (23) |  | 29.0  (47) |  | 46.6  (76) |  |
| Missing | 40.9  (9) | 30.44*** | 23.8  (5) | 37.21*** | 45.5  (5) | 10.38* | 10.0  (1) | 13.58** | 36.4  (4) | 21.29** | 36.4  (4) | 26.15*** |
|  |  |  |  |  |  |  |  |  |  |  |  |  |
| Bullied in grade 11  (age 17-18) |  |  |  |  |  |  |  |  |  |  |  |  |
| No | 23.8  (595) |  | 25.7  (645) |  | 22.7  (238) |  | 16.1  (169) |  | 24.5  (357) |  | 32.7  (476) |  |
| Yes | 43.7  (66) |  | 42.4  (64) |  | 36.7  (18) |  | 22.5  (11) |  | 47.1 (48) |  | 52.0  (53) |  |
| Don’t know | 40.6  (58) |  | 37.8  (54) |  | 30.3  (20) |  | 31.8  (21) |  | 49.4  (38) |  | 42.9 (33) |  |
| Missing | 25.9  (134) | 47.02*** | 24.5  (127) | 30.35*** | 23.5  (66) | 6.77 | 16.3 (46) | 11.99** | 28.8 (68) | 44.77*** | 34.2 (81) | 18.28*** |

***p<0.001 **p<0.01 *p<0.05

Supplementary Material. Table S3. Results from binary logistic regression analyses predicting depressive and anxiety symptoms at age 20-21 by exposure to bullying in grades 9 (age 15-16) and 11 (age 17-18). Models fully adjusted for bullying, gender, family type, parental education, parental country of birth, and medication for depression and anxiety. Wald tests from separate models that include interaction terms between bullied and gender. n=2323

|  | Depression symptoms | | Anxiety symptoms | |
| --- | --- | --- | --- | --- |
|  | OR | 95% CI | OR | 95% CI |
| Bullied |  |  |  |  |
| Neither in grade 9 nor 11 | 1.00 | - | 1.00 | - |
| In grade 9 only | 1.59* | 1.04-2.43 | 1.80** | 1.20-2.69 |
| In grade 11 only | 1.74* | 1.05-2.89 | 1.50 | 0.88-2.55 |
| In both grade 9 and 11 | 2.49** | 1.39-4.46 | 1.85* | 1.05-3.29 |
|  |  |  |  |  |
| Gender |  |  |  |  |
| Males (ref.) | 1.00 | - | 1.00 | - |
| Females | 1.05 | 0.85-1.30 | 2.27*** | 1.82-2.83 |
|  |  |  |  |  |
| Family type |  |  |  |  |
| Two original parents (ref.) | 1.00 | - | 1.00 | - |
| One parent | 1.52** | 1.15-2.01 | 1.45* | 1.09-1.94 |
| Shared residence | 1.11 | 0.85-1.45 | 1.34* | 1.02-1.76 |
| Other | 1.68 | 0.89-3.17 | 1.62 | 0.92-2.85 |
|  |  |  |  |  |
| Parental education |  |  |  |  |
| ≤2 years secondary or less | 1.19 | 0.85-1.67 | 1.24 | 0.87-1.77 |
| ≥3 years secondary (ref.) | 1.00 | - | 1.00 | - |
| Tertiary | 0.96 | 0.74-1.25 | 1.05 | 0.80-1.38 |
|  |  |  |  |  |
| Parental country of birth |  |  |  |  |
| At least one in Sweden (ref.) | 1.00 | - | 1.00 | - |
| Two parents outside Sweden | 1.67*** | 1.29-2.16 | 1.39* | 1.05-1.83 |
|  |  |  |  |  |
| Medication for depression | 2.57** | 1.44-4.57 | 1.55 | 0.90-2.66 |
|  |  |  |  |  |
| Medication for anxiety | 1.36 | 0.80-2.30 | 1.92* | 1.15-3.18 |
|  |  |  |  |  |
| *Wald test for interaction bullied*gender* | *χ^2^=2.28* | *p=0.516* | *χ^2^=2.99* | *p=0.393* |

***p<0.001 **p<0.01 *p<0.05
